# Distinct Modes of Transmission of Tuberculosis in Aboriginal and Non-Aboriginal Populations in Taiwan

**DOI:** 10.1371/journal.pone.0112633

**Published:** 2014-11-13

**Authors:** Yih-Yuan Chen, Fan-Chen Tseng, Jia-Ru Chang, Shu-Chen Kuo, Jen-Jyh Lee, Jun-Jun Yeh, Tzong-Shi Chiueh, Jun-Ren Sun, Ih-Jen Su, Horng-Yunn Dou

**Affiliations:** 1 National Institute of Infectious Diseases and Vaccinology, National Health Research Institutes, Zhunan, Miaoli, Taiwan; 2 Graduate Institute of Basic Medical Science, China Medical University, Taichung, Taiwan; 3 Department of Microbiology, Hualien Tzu Chi Medical Center, Hualien, Taiwan; 4 Ditmanson Medical Foundation Chia-Yi Christian Hospital, Chiayi, Taiwan; 5 China Nan University of Pharmacy and Science, Tainan, Taiwan; 6 Meiho university, Pingtung, Taiwan; 7 Department of Medical Research, Pingtung Christian Hospital, Pingtung, Taiwan; 8 Chung Shan Medical University, Taichung, Taiwan; 9 Division of Clinical Pathology, Department of Pathology, Tri-Service General Hospital and National Defense Medical Center, Taipei, Taiwan; Chang-Gung University, Taiwan

## Abstract

Tuberculosis incidence among aborigines is significantly higher than for Han Chinese in Taiwan, but the extent to which *Mycobacterium tuberculosis* (MTB) strain characteristics contribute to this difference is not well understood. MTB isolates from aborigines and Han Chinese living in eastern and southern Taiwan, the major regions of aborigines, were analyzed by spoligotyping and 24-loci MIRU-VNTR. In eastern Taiwan, 60% of aboriginal patients were ≤20 years old, significantly younger than the non-aboriginal patients there; aborigines were more likely to have clustered MTB isolates than Han Chinese (odds ratio (OR) = 5.98, p<0.0001). MTB lineages with high clustering were EAI (54.9%) among southern people, and Beijing (62.5%) and Haarlem (52.9%) among eastern aborigines. Resistance to first-line drugs and multidrug resistance (MDR) were significantly higher among eastern aborigines (≥15%) than in any other geographic and ethnic group (p<0.05); MDR was detected in 5 of 28 eastern aboriginal patients ≤20 years old. Among patients from the eastern region, clustered strains (p = 0.01) and aboriginal ethnicity (p = 0.04) were independent risk factors for MDR. The lifestyles of aborigines in eastern Taiwan may explain why the percentage of infected aborigines is much higher than for their Han Chinese counterparts. The significantly higher percentage of the MDR-MTB strains in the aboriginal population warrants close attention to control policy and vaccination strategy.

## Introduction

Tuberculosis (TB) remains a global threat to public health and is considered by the World Health Organization (WHO) to be an epidemic [1]. It is still the leading cause of death for notifiable infectious diseases in Taiwan despite a steady decrease in both TB incidence and mortality rates since 1950. Currently, 2.26% of the inhabitants of Taiwan are aboriginal people (Statistics of Council of Indigenous Peoples, Executive Yuan, Taiwan). Notably, aboriginal populations experience a 4- to 6-fold higher incidence and prevalence of TB compared to non-aboriginal populations [2]. The reasons for this are not fully understood, but could include a variety of factors such as predisposing genetic polymorphisms of the human *SLC11A1* (NRAMP-1) gene [3] and the particular *Mycobacterium tuberculosis* (MTB) strains circulating in the respective populations, as well as societal issues. Eastern Taiwan is mountainous and the least developed region of the island, and the aborigines residing there have relatively primitive lifestyles, lower socio-economic status, and reduced access to health care [4]. Although most aborigines in southern Taiwan also have intact village lifestyles, they have better transportation and living conditions as compared to their eastern counterparts. According to reports from the Taiwan Centers for Disease Control in 2010, the eastern region experienced an annual incidence and sputum positive rate of TB of 99.7 and 48.3 cases per 100,000 inhabitants, significantly higher than in southern region (76.4 and 29.3 cases per 100,000 inhabitants). To better understand the reasons for the high TB rates among aborigines, we investigated the genetics of MTB strains infecting aboriginal and Han Chinese TB patients in eastern Taiwan, and used TB patients from southern Taiwan as the comparison group.

The purpose of this study was twofold: (1) to compare the genotypes of MTB lineages and their genetic clustering in aboriginal and Han Chinese populations of eastern Taiwan and southern Taiwan, two geographic regions where relatively high numbers of aborigines live; (2) to determine the prevalence of and factors associated with drug-resistant MTB strains in the aforementioned groups. We analyzed several hundred MTB strains by spoligotyping, MIRU-VNTR typing [5], [6], [7] and drug susceptibility testing. Results of a previous study by our group indicated that Beijing and Haarlem strains are the predominant MTB strains in aborigines in eastern Taiwan, whereas EAI and Haarlem strains are the predominant strains among aborigines in southern Taiwan [5]. However, the transmissibility of MTB strains in aborigines and non-aborigines is not well understood. Strain surveillance analysis, together with drug resistance studies, will help to pinpoint isolates associated with higher prevalences of MTB, with the aim of directing efforts to limit the spread of those strains within aboriginal and Han Chinese communities.

## Materials and Methods

### Study setting

Major areas of aborigine habitation are located in eastern and southern Taiwan, and thus large referral hospitals in each region were selected as sampling sites, Kaohsiung Chang-Gung Memorial Hospital (KCGH) in the south and Tzu-Chi General Hospital (TCGH) in the east. KCGH is the largest tertiary hospital in Kaohsiung, with 2700 beds, and TCGH is the only tertiary referral hospital in eastern Taiwan. From January 2005 to December 2008 (eastern) and January 2010 to December 2011 (southern), 148 (eastern) and 185 (southern) consecutive patients with symptoms compatible with pulmonary TB and with sputum cultures positive for *M. tuberculosis* complex were included in the study. All isolates were genotyped by spacer oligonucleotide typing (spoligotyping) and 24-locus MIRU-VNTR typing. The *M. tuberculosis* strain H37Rv was used as the control. This study was approved by the Human Ethics Committee of the National Health Research Institutes, Taiwan (Code: EC1010804-E). Because of the retrospective nature, routine collection of minimal clinical data in hospital laboratories, and dislinkage of personal information, the requirement to obtain informed consent from each patient was waived by the NHRI institutional review board. The patient record/information was anonymized and de-identified prior to analysis.

### Spoligotyping and spoligotyping analysis

Spoligotyping was carried out according to the manufacturer’s instructions (Isogen Bioscience B.V., Maarsen, The Netherlands). The SITVITWEB database [6] and a web-based computer algorithm, Spotclust [7], were used to assign new isolates to families, subfamilies and variants.

### 24-locus MIRU-VNTR typing

The 12 classical MIRU-VNTR loci (‘12-locus’), 3 exact tandem repeats (ETR A, B and C) and 9 additional loci (Mtub04, Mtub21, Mtub29, Mtub30, Mtub34, Mtub39, QUB11b, QUB26 and QUB4156) were individually amplified in all MTB isolates as previously described by Supply et al. [8]. The resulting typing pattern was used to create a 24-digit allelic profile for each isolate, and isolates with all 24 loci identical were defined as belonging to the same cluster.

### Drug susceptibility test

Drug susceptibility testing was performed according to the Clinical and Laboratory Standards Institute (CLSI) standard [9]. Tests were conducted by using the agar proportion method utilizing Middlebrook 7H10 agar supplemented individually with the following drugs: ethambutol (5 and 10 µg/ml), isoniazid (0.2 and 1 µg/ml), rifampicin (1 µg/ml) and streptomycin (2 and 10 µg/ml).

### Statistical analysis

Within each geographic region and ethnic population, all MTB isolates were characterized based on the 24-loci typing results, and the numbers of total (T), unique (U) and clustered isolates (C), and clusters (N). Clustering rate (C% = C/T) was calculated as previously defined [10]. Fisher’s exact test and odds ratio (OR) with 95% confidence interval (95% C.I.) were used to express the significance and magnitude, respectively, of the associations between strain clustering, demographics, ethnicity, MTB genotypes and drug resistance.

## Results

### Characteristics of TB patients

At TCGH (eastern Taiwan), 148 MTB isolates from 47 aboriginal patients (31.76%) and 101 non-aboriginal patients (Han Chinese) (68.24%) were subjected to spoligotyping and MIRU-VNTR typing. The aboriginal patients in this sample were significantly younger than the Han Chinese patients: nearly 60% of the former were less than 20 years old, whereas 78.21% of the non-aboriginals were older than 41 years (Table 1). At KGCH (southern Taiwan), 185 MTB isolates from 43 aboriginal patients (23.24%) and 142 Han Chinese patients (76.76%) were examined. In contrast to TCGH, the age distributions for the two ethnic groups in the KGCH sample did not differ (p = 0.6; Table 1), and around half of the patients were older than 70 years. When all patients were divided into 4 groups according to geography (southern vs. eastern Taiwan) and ethnicity (aborigine vs. Han Chinese), the percentage of females in the eastern aborigine group was slightly higher (46.8%; Table 1) than in the other three groups (≤33.8%; p = 0.09).

**Table 1:** Characteristics of the tuberculosis (TB) patients from whom the study’s *Mycobacterium tuberculosis* strains were isolated.

	No. (%) of TB cases			
	Eastern		Southern	
	Aborigine	Han Chinese	Aborigine	Han Chinese
**Total**	47(37.76)	101(68.24)	43(23.24)	142(76.76)
**Sex**				
Male	25(53.19)	74(73.27)	31(72.09)	94(66.2)
Female	22(46.81)	27(26.73)	12(27.91)	48(33.8)
**Age**				
Average	29.43	56.16	65	67.95
≦20 years	28(59.57)	10(9.90)	0(0.00)	1(0.7)
21–40 years	2(4.26)	12(11.88)	5(11.63)	10(7.04)
41–70 years	16(34.04)	50(49.50)	19(44.19)	56(39.44)
>70 years	1(2.13)	29(28.71)	19(44.19)	75(52.82)

Gender and age distribution between aborigines and Han Chinese were significantly different for the eastern region (p = 0.024 and p<0.0001, respectively), but not for the southern region (p = 0.58 and p = 0.6, respectively).

### Predominant *M. tuberculosis* genotypes in eastern and southern regions of Taiwan

Molecular analysis showed that, in eastern Taiwan, the Beijing lineage (51.06% in aborigines, 44.55% in Han Chinese) was the most prevalent (Table 2), followed by Haarlem (36.17% in aborigines, 21.78% in Han Chinese) and T lineages (10.64% in aborigines, 13.86% in Han Chinese). No EAI strain was isolated from eastern aboriginal patients (Table 2; Fig. S1A), whereas EAI strains were isolated from 8.8% of eastern Han Chinese patients (Table 2). In the south, the most prevalent genotype in the two ethnic groups was EAI (46.51% in aborigines, 43.66% in Han Chinese) (Fig. S1B, followed by Haarlem (20.93%) in aborigines and Beijing (27.46%) in Han Chinese (Table 2). A similarity between the two regions is that the Haarlem genotype, although not the predominant strain, was more prevalent in the respective aboriginal groups (Table 2).

**Table 2:** Distribution of strain lineages among MTB isolates in aboriginal and Han Chinese patients from eastern and southern Taiwan.

	No. (%) of strains in each ethnic population						
	Eastern			Southern			
	Total	Aborigine	Han Chinese		Total	Aborigine	Han Chinese
**Beijing**	69(46.62)	24(51.06)	45(44.55)		47(25.41)	8(18.6)	39(27.46)
**EAI**	9(6.08)	0(0)	9(8.91)		82(44.32)	20(46.51)	62(43.66)
**Haarlem**	39(26.35)	17(36.17)	22(21.78)		28(15.14)	9(20.93)	19(13.38)
**T**	19(12.84)	5(10.64)	14(13.86)		19(9.73)	5(11.63)	13(9.15)
**LAM**	3(2.03)	0(0)	3(2.97)		2(1.08)	1(2.33)	1(0.7)
**Others**	9(6.08)	1(2.13)	8(7.92)		8(4.38)	0(0)	8(5.63)

### MTB clustering and transmission associated with ethnic groups and strain lineages

We examined if there is a relationship between strain clustering, which was defined as sharing the same spoligotyping and 24-MIRU-VNTR result, and demography, ethnicity or geographic region. The overall MTB-clustering rates were similar between strains from the east (26.35%) and the south (32.43%; p = 0.28), but distributions differed by ethnicity. Eastern aboriginal patients showed a significantly higher clustering rate (51.06%) than Han Chinese patients (14.85%), which yielded an odds ratio (OR) of 5.98 (95% CI = 2.71–13.21, p<0.0001; Table 3).

**Table 3:** Association between cluster rates and *M. tuberculosis* genotype in aboriginal and Han Chinese populations.

		Whole Population					Beijing					EAI					Haarlem				
Region	Group	No. ofpatients	Patients incluster (%)	OR	95% CI	Pvalue	No. ofpatients	Patientsin cluster (%)	OR	95% CI	Pvalue	No. ofpatients	Patientsin cluster(%)	OR	95% CI	Pvalue	No. ofpatients	Patientsin cluster(%)	OR	95% CI	Pvalue
**Eastern**	**Total**	148	39(26.35)				69	24(34.78)				9	2(22.22)				39	13(33.33)			
	**Aborigine**	47	24(51.06)	5.98	2.71–13.21	<0.0001	24	15(62.50)	6.67	2.21–20.09	0.001	0	0	NA	NA	1	17	9(52.94)	5.06	1.2–21.42	0.04
	**Han Chinese**	101	15(14.85)	**Ref**			45	9(20.00)	**Ref.**			9	2(22.22)	**Ref.**			22	4(18.18)	**Ref.**		
**Southern**	**Total**	185	60(32.43)				47	8(17.02)				82	45(54.88)				28	4(14.29)			
	**Aborigine**	43	10(23.26)	0.56	0.25–1.22	0.14	8	0(0)	0.22*	0.01–4.17*	0.32	20	8(40.00)	0.45	0.16–1.26	0.2	9	2(22.22)	2.43	0.28–20.82	0.57
	**Han Chinese**	142	50(35.21)	**Ref**			39	8(20.51)	**Ref.**			62	37(59.68)	**Ref.**			19	2(10.53)	**Ref.**		

*These logit estimators use a correction of 0.5 in every cell that contains a zero.

We investigated whether the MTB genotypes involved in clustering differed by ethnic group or region, which might suggest differences in genotype transmission fitness or founder effects in each population. The highest clustering rates were detected for the Beijing lineage (34.78%) in the east and the EAI lineage (54.88%) in the south (Table 3). In the east, the percentages of clustered isolates of the Beijing and Haarlem lineages were significantly higher among aborigines (62.5% and 52.94% for Beijing and Haarlem, respectively; Table 3) than among the Han Chinese (20% and 18.18%, respectively), and EAI and its clustering was detected only in Han Chinese (22.22%). However, in the south, the EAI and Beijing genotypes had no significant differences among aborigines (40% and 0% for EAI and Beijing, respectively) and Han Chinese (59.68% and 20.51%, respectively).

In the two regions, the results of analysis of MTB-isolate clustering within and across ethnic groups are as follows. In eastern Taiwan, 5 and 5 clusters were detected exclusively within aborigines (3 Beijing and 2 Haarlem) and Han Chinese (3 Beijing, 1 EAI and 1 Haarlem), respectively, and 4 (2 Beijing and 2 Haarlem) were across ethnic groups; 3 clusters of larger sizes (involving more than 5 patients) were all detected exclusively from aborigines (Table S1A). In southern Taiwan there were 13 clusters exclusively within Han Chinese (4 Beijing, 8 EAI and 1T), none exclusively from aborigines, and 8 (6 EAI and 2 Haarlem) across ethnic groups (Table S1B). Three larger clusters, 1 exclusively in Han Chinese and 2 across ethnic groups, were detected in the south.

In addition, we compared patient age distributions in relationship to clustering. In the east, patients with clustered strains were significantly younger than those with unique strains (median = 41 vs. 53, p = 0.007 by Wilcoxon 2-sample Test), but this was not the case in the south (median = 68.5 vs. 72, p = 0.17). Furthermore, the clustered strains in the east (43.6%) were identified from individuals younger than 20 years old, while in the south (45%) they originated from patients older than 70 years. In the east, 5 of 8 Beijing and 5 of 5 Haarlem MTB clusters involved patients younger than 20 years; 54% of the 24 eastern aborigines who had clustered strains were less than 20 years old. In contrast, among this young group in the south, only 1 Han Chinese had a clustered strain of the T lineage. In fact, 43% and 45% of the clustering in the south were detected among patients more than 70 years old and between 41–70 years old, respectively, while the corresponding percentages in the east were 38.46% and 12.82%. In summary, in the east, MTB strain-clustering was found mostly among individuals ≤20 years of age, but in the south the clustering was much more frequent among older patients.

### Drug resistance and multidrug resistant-TB: a severe problem in eastern aborigines

Drug resistance patterns of the MTB strains also differed by region and ethnicity, and, of the four groups, the eastern aborigines had the highest resistance to all first-line anti-TB agents. The percentages of strains resistant to each of isoniazid, ethambutol and rifampicin and multidrug resistance (MDR; defined as resistant to at least isoniazid and rifampicin) were significantly higher among aborigines than Han Chinese in the eastern region (p≤0.05) but did not differ between the two ethnic groups in the south (Table 4). In the east, aborigines were much more likely than Han Chinese to have MDR MTB (OR = 11.7, 95% C.I. = 2.42–56.76; p<0.0001), but no association (p = 1) was found in the south. Our study found 9 of 11 MDR-TB were in clusters from the eastern region. Notably, 3 of the 9 MDR-TB strains were clustered with non-MDR TB strains, and all were isolated from teenagers (2 of 3 were aborigines). Eight of 9 MDR strains from eastern aborigines were of the Beijing genotype, and notably 6 of them were from the same cluster that involved two teenagers and four adults over the age of 41. Surprisingly, another 3 MDR strains among eastern aborigines were also isolated from teenagers. In terms of genotype association with MDR in both regions, the Beijing (7.76%) lineage had the highest rate of MDR, followed by the Haarlem lineage (2.99%; p = 0.33, compared to Beijing) and EAI (1.1% p = 0.05, compared to Beijing). In multivariable analyses of patients from the eastern region, having a clustered strain (p = 0.01) and aboriginal ethnicity (p = 0.04) were independently associated with higher risks of MDR (OR = 8.91 and 5.65, respectively).

**Table 4:** Drug susceptibility and resistance to first-line anti-tuberculosis drugs.

	Eastern		Southern		
	Aborigines (n = 47)	Non-aborigines (n = 101)		Aborigines (n = 43)	Non-aborigines (n = 142)
**All sensitive**	31(65.96)	80(79.21)		38(88.37)	113(79.58)
**Drug resistant**					
**Streptomycin**	10(21.28)	16(15.84)		3(6.98)	13(9.15)
**Isoniazid**	12(25.53)	12(11.88)*		2(4.65)	18(12.68)
**Ethambutol**	7(14.89)	0(0)*		1(2.33)	1(0.7)
**Rifampicin**	10(21.28)	2(1.98)*		1(2.33)	1(0.7)
**MDR** a	9(19.15)^a^	2(1.98)* ^b^		0(0)	1(0.7)^c^

*P≤0.05 by Fisher exact test comparing drug-resistance percentages between aborigine and non-aborigine populations.

a MDR: resistant to at least isoniazid and rifampicin.

## Discussion

The present study provides profiles of MTB genotypes and drug resistance patterns among isolates from aborigines and Han Chinese living in eastern and southern Taiwan, the major regions of aborigines on the island.

The lifestyles of aborigines in eastern Taiwan may explain why the infected aborigines were much younger than their Han Chinese counterparts (Table 1). Aboriginal people in eastern Taiwan have fewer opportunities for employment there, causing those of working age (ages 20–55) to move to more developed areas. Thus, the young adults and children remaining in the village have closer interactions with their grandparents, who may be TB-infected/relapsed patients, thus contributing to the high TB prevalence observed among eastern aborigines less than 20 years old. This hypothesized transmission route needs to be investigated through contact tracing. Notably, some of the infected children or teenagers might also have transmitted MTB to their peers, as we observed few clusters that involved teenagers of both aboriginal and Han Chinese ethnicity.

Among the aborigines in eastern Taiwan, intra-community transmission of MTB seemed to account for a substantial proportion of the TB endemicity and associated drug-resistance problems. The most effective means to prevent TB transmission relies upon case-finding and treatment to eliminate sources of infections. Unfortunately, anti-TB drugs encounter resistant MTB. A decreasing trend of anti-TB drug resistance in MTB has been reported in Taiwan [11]. However, our results show that the eastern aboriginal sample population comprises 31.76% of the total eastern sample population (47/148), and 81.82% of MDR-TB patients were aborigines (9/11) (Table 4). These results coincide with previous studies reported by Hsu et al. [12]. Our results also show a significantly higher frequency of resistance to anti-TB drugs in eastern aboriginal patients when compared to Han Chinese (Table 4). The high percentage and transmission rates of MDR-TB in aborigines indicate that MDR-TB is an emergent problem in the eastern aboriginal population. Surprisingly, 6 of 11 (54.55%) eastern MDR-TB strains were isolated from teenagers (5 of 6 teenagers were aborigines). The development of clustered stains from non-MDR-TB strains to MDR-TB strains may be due to discontinuous treatment. Taken together, the transmission of MDR-TB, especially among aborigine teenagers, should receive closer attention.

The EAI lineage, the most ancient MTB lineage [13], is prevalent in Southeast Asia, particularly in the Philippines (73%), in Myanmar and Malaysia (53%), and in Vietnam and Thailand (32%) [14]. Chang et al. reported that EAI strains are highly prevalent in southern Taiwan [15]. In the present study, we also found EAI strains to be prevalent in the two studied ethnic groups of southern Taiwan. It is a very interesting question why EAI strains are so prevalent in southern Taiwan. The climate in southern Taiwan is similar to that of the Philippines and Malaysia. EAI strains are prevalent in countries with tropical climates, suggesting that EAI strains may be better adapted for growth and transmission in high-temperature environments, but this remains to be determined.

Our study showed that Beijing lineages are the most prevalent strains in eastern Taiwan, whereas in southern Taiwan they are the second most prevalent strains (Table 2). Beijing strains originated in China and are now dominant not only in that country (82%), but also in neighboring countries such as South Korea (91%), Japan (79%), Vietnam (53%), Thailand (44%) and Indonesia (44%) [16], [17], [18], [19], [20], [21], [22]. Previous studies indicated that, in eastern, northern and southern Taiwan, the most prevalent strains belonged to the Beijing lineage, followed by Haarlem and EAI [23]. The present study showed EAI to be the most prevalent strain in southern Taiwan (Table 2), which could be due to the higher transmissibility of the EAI strains (54.88%) compared to Beijing strains (17.02%) in southern Taiwan (Table 3).

We observed several differences in MTB isolate-clustering patterns by region and ethnicity. First, isolate clustering in the east was more frequent among aborigines. This concurs with the cluster size findings: in the east, the larger clusters (involving >4 patients) all comprised MTB isolates from aborigines, but in the south the larger clusters were more likely from Han Chinese. Second, the EAI lineage had an unusually high prevalence and clustering in the southern Taiwan sample, especially striking since the Beijing lineage is known to be the most prevalent MTB in Taiwan. Third, among the four geographic-ethnic populations, the southern aboriginal group was the only one in which we did not detect within-group clustering, which suggests that chain-transmission of MTB within this population was not as severe as for the other three populations. This finding may indicate that aborigines in southern Taiwan experience more frequent interactions with the Han Chinese than do their eastern aboriginal counterparts, probably due to differences in lifestyles such as convenient mass transportation in the south.

This retrospective study had some limitations. We hope to initiate a prospective collection of MTB strains and patient information to validate the strain differences based on ethnicity, region, lineage and clustering we observed in the present study. Finally, because this was a hospital-based study, TB patients with apparent or more severe symptoms would be more likely to attend or be referred to the two hospitals, and we expect that hospital admittance would be less likely in the eastern region, where medical resources are more limited.

Strain analysis, together with epidemiological surveillance, will also help in pinpointing isolates associated with higher morbidity and mortality, with the aim of directing efforts to limit the spread of those strains within aboriginal and Han Chinese communities. The high prevalence of the Beijing genotype in the aboriginal population warrants close attention to control policy and vaccination strategy.

## Supporting Information

Figure S1 **A minimum spanning tree based on 24–MIRU-VNTR genotyping of 148 MTB isolates in eastern (A) and southern (B) Taiwan.** The sizes of the branches correspond to the number of isolates with a particular genotype. Indo-oceanic lineage (EAI), East-Asia lineage (Beijing) and Euro-American lineage (Haarlem). The MTB strains from aboriginal and Han Chinese patients are assigned different colors.
(TIF)

Table S1 **A.** Characterization of strain lineages amongst MTB isolates in aboriginal and Han Chinese patients from eastern Taiwan. **B.** Characterization of strain lineages amongst MTB isolates in aboriginal and Han Chinese patients from southern Taiwan.
(XLSX)
