# Supplementary figures and images for: Distinct Modes of Transmission of Tuberculosis in Aboriginal and Non-Aboriginal Populations in Taiwan
Source: PLoS One. 2014 Nov 13;9(11):e112633. doi: 10.1371/journal.pone.0112633 (PMC4231046; doi:10.1371/journal.pone.0112633)

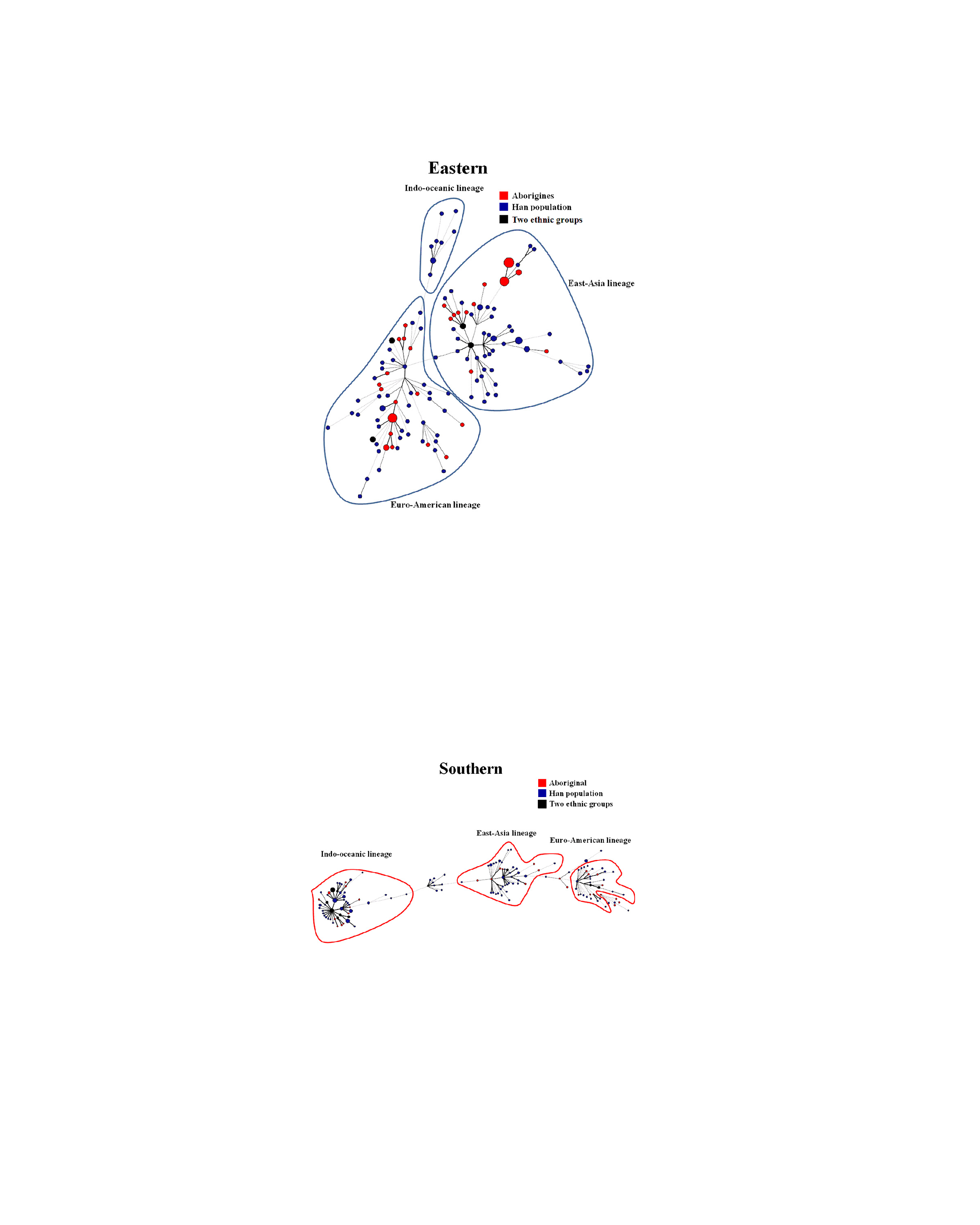

Supplement: Figure S1 — A minimum spanning tree based on 24–MIRU-VNTR genotyping of 148 MTB isolates in eastern (A) and southern (B) Taiwan. The sizes of the branches correspond to the number of isolates with a particular genotype. Indo-oceanic lineage (EAI), East-Asia lineage (Beijing) and Euro-American lineage (Haarlem). The MTB strains from aboriginal and Han Chinese patients are assigned different colors. (TIF) [file pone.0112633.s001.tif]
